# Myths Pertaining to COVID-19 Vaccination in Pregnant Women Attending a Rural Tertiary Care Hospital

**DOI:** 10.7759/cureus.57112

**Published:** 2024-03-28

**Authors:** Harinder Kaur, Monika Jindal, Sameer Singh Faujdar, Santosh Minhas, Nitin Rathi, Navneet Kaur

**Affiliations:** 1 Obstetrics and Gynaecology, Maharishi Markandeshwar Medical College and Hospital, Solan, IND; 2 Microbiology, Maharishi Markandeshwar Medical College and Hospital, Solan, IND

**Keywords:** vaccine, pregnancy, neonate, myths, fetus, covid-19

## Abstract

Background

The outbreak of the COVID-19 pandemic led to the rise of various social issues apart from medical ones. Several myths regarding COVID-19 vaccination were found worldwide, and some of the common ones identified were abortions, birth defects, bad pregnancy outcomes such as abortions, ectopic pregnancy, risk of infertility, and irregular menstrual cycles. Although no scientific theories or data backed those myths, pregnancy was still omitted from trials for a long time as any drug/vaccine given during pregnancy may affect the fetus.

Objective

The objective of this study was to evaluate the vaccination status of pregnant women (PW) regarding COVID-19 and explore the factors influencing those who chose not to get the initial dose, second dose, or booster dose.

Methodology

A total of 747 PW were enrolled in the current study. Information related to sociodemographic data, clinical data, COVID-19 vaccine status, and rationale for choosing not to receive the COVID-19 vaccination was analyzed using a prestructured and validated Performa.

Results

The mean age and gestational age of the women enrolled for the study was 27.39 ± 3.75 years and 30.21 ± 7.30 weeks, respectively. The first dose of the COVID-19 vaccine was not received by 40 (5.4%) subjects, and the second dose was pending in 142 (19%) women, and none of them received booster dose. The prevalent cause for abstaining from receiving the COVID-19 vaccination was the fear of abortion in 179 (24%) subjects, followed by the fear of vaccine-related side effects in 142 (19%) subjects. There was a significant correlation between acceptance of COVID-19 vaccination and education and employment.

Conclusion

The present study indicated that most women have taken the COVID-19 vaccine before conception and that none received the first, second, or booster dose during pregnancy, even if it was due. Women need to be educated about the benefits of vaccination to enhance the compliance rate of COVID-19 vaccination and reduce COVID-19-related morbidity and mortality during pregnancy.

## Introduction

COVID-19 was declared a global pandemic on March 11, 2020, by WHO. To date, it is difficult to determine the exact burden of pregnant women (PW) who have suffered from COVID-19 [1]. PW infected with COVID-19 have been observed to have a higher likelihood of experiencing premature births, cesarean deliveries, and other unfavorable pregnancy outcomes such as stillbirths, maternal fatalities, ruptured extrauterine pregnancies, and maternal depression [2]. Until August 19, 2021, there were 105,645 PW in the USA who had COVID-19, out of which 18,008 were hospitalized, and 124 suffered fatalities among hospitalized PW, which also had a negative impact on the general health of expectant mothers and their unborn children [2,3]. Despite COVID-19 being a novel disease, data showed that PW were likely to encounter more severe symptoms. According to most recent studies, PW with COVID-19 required more extensive treatment as compared to non-PW [3]. There was a consistent link between COVID-19 in pregnancy and a high incidence of unfavorable outcomes. Therefore, immunization against COVID-19 was advised for everyone, including PW, nursing mothers, and women who were keen for conception in the future [4]. The benefits of obtaining a COVID-19 vaccine appeared to outweigh any known or prospective risks of immunization during pregnancy [4]. There are no data that state that COVID-19 vaccinations harm female fertility, and recent findings confirm their immunogenicity and efficacy [4]. On July 2, 2021, the Union Health Ministry of India approved COVID-19 vaccination for PW and said that vaccination can be given at any time during pregnancy, taking into consideration the risks and benefits [5]. Pregnancy, being an immunocompromised state, makes PW more susceptible to infectious diseases such as COVID-19 infection. PW’s immune system does not maintain a static immunosuppressed state, rather the immunological condition changes along with the fetus's development. In early pregnancy, the maternal immune system is pro-inflammatory, which favors the implantation of the embryo and placentation. In the middle half of pregnancy, it is anti-inflammatory, which helps fetal growth, while near the delivery of the baby, it again becomes pro-inflammatory, which favors the parturition [6]. COVID-19 vaccination is strongly advised for PW by prestigious medical organizations such as the American College of Obstetricians and Gynecologists (ACOG), American Academy of Family Physicians, American Academy of Pediatrics (AAP), and American-College of Nurse-Midwives, Federation of Obstetrics and Gynecological Societies of India (FOGSI). Furthermore, although it is not feasible to vaccinate newborns, COVID-19 immunization develops antibodies that provide fetal and infant protection against the illness. Hence, even if they are not yet eligible, the infant will already be immune to dangerous illness by passive immunity [6,7]. The COVID-19 vaccine is advised during pregnancy to prevent maternal morbidity, mortality, and several unfavorable birth outcomes. Globally, each nation has its own laws regarding COVID-19 immunization during pregnancy. Out of 91 nations that acknowledged the policy, 45 countries allow the immunization of PW [3]. Several myths and obstacles prevent PW from receiving the COVID-19 vaccine. The present study aimed to analyze the COVID-19 vaccination status among the PW and to investigate the reasons behind not receiving the COVID-19 vaccine during pregnancy despite the guidelines issued by the Health Ministry of India.

## Materials and methods

Study design: Single-center hospital-based prospective study was conducted from July 2022 until June 2023 at a tertiary-care hospital situated in the Solan district after approval from the institutional ethical committee (MMMCH/IEC/22/553). A total of 747 PW irrespective of their gestational age were enrolled in the study by random sampling who have visited the obstetrics and gynecology department for antenatal checkup and were found to fit into the inclusion criteria. Information related to sociodemographic data such as age; residential area; occupation and education status; clinical data concerning their last menstrual period; period of gestation; any significant obstetrical, medical, and surgical history in the past; and COVID-19 vaccine status whether received or not, if yes, then the number of doses received were enquired. Reasons behind the non-reception of the COVID-19 vaccine (if not received previously or incomplete vaccination) during pregnancy were analyzed using a prestructured and prevalidated Performa.

Inclusion criteria: All PW of the reproductive age group (15-45 years) who have taken either incomplete vaccination including before conception and have not taken other doses because of pregnancy or did not opt for COVID-19 vaccine dose at all until this date, even after clear guidelines for COVID-19 vaccination in pregnancy and lactation were provided by the government of India.

Exclusion criteria: Fully vaccinated PW with the COVID-19 vaccine, subjects unwilling to participate in the study, those mentally unstable, and patients coming into active labor were excluded from the study.

Method of data collection: The questionnaire was designed and developed specifically for this study by utilizing questions that were necessary for establishing the sociodemographic data, clinical data, COVID-19 vaccine status of the study subjects, and their opinions regarding vaccination. Individual interviews of patients coming to the outpatient department (OPD) were conducted to know the status of the vaccine doses received and the myths behind vaccine hesitancy and to assess the knowledge and awareness about COVID-19 vaccination in PW. This interview was voice recorded for better understanding and prompt analysis.

Participants were well-informed about the objective of the study and handling of their personal information. Informed consent was taken in writing, and data were kept anonymous.

Statistical analysis: Data were captured on the physical proforma, and a master chart was prepared after the completion of the study on a Microsoft Excel sheet and analyzed using Statistical Product and Service Solutions (SPSS) (IBM SPSS Statistics for Windows, Armonk, NY). For quantitative variables, mean and standard deviation were calculated. For qualitative variables, the number and proportions of the total were calculated. Tables were used to depict the data. P values calculated by the student T-test and P values of <0.05 were considered significant and values >0.05 were considered nonsignificant.

## Results

The mean age of the study for women was 27.39 ± 3.75 years. A notable significant value was observed in the vaccination status concerning education (p=0.0001) and employment (p=0.005). Among the total of 747 subjects, 401 (53.7%) had an education level of below secondary education and 249 (33.3%) were graduates, out of which 42.8% and 4% were not vaccinated, respectively. Approximately 86% of the PW were homemakers, and ≈ 27% of them were not vaccinated. The vaccination rate was significantly higher in the educated (P value=0.0001) and employed categories (P value=0.005), and 12% of the employed population (total=102) was not vaccinated. Those not vaccinated among the employed category were unskilled workers. The findings revealed that individuals with higher levels of education and those employed in skilled positions were more inclined to receive vaccination (Table 1).

**Table 1:** Sociodemographic data of study subjects *: Significant P value

Variable	Subdomain	n (%)	Not vaccinated	P value
Education	≤12th	401 (53.7%)	173 (43%)	0.0001*
	Graduate	249 (33.3%)	10 (4%)	
	Postgraduate	82 (11%)	00	
	Doctorate	15 (2%)	00	
Employment	Homemakers	640 (85.7%)	170 (26.5%)	0.0059*
	Skilled workers	102 (13.6%)	12 (12%)	
	Unskilled workers	5 (0.6)	4 (80%)	

The mean gestation period was 30.21 ± 7.30 weeks. Among the total 747 enrolled subjects, 114 (15.3%) were in the first trimester, 123 (16.5%) and 510 (68.3%) in the second and third trimesters, respectively, and only five patients received their due dose of the vaccine in the third trimester (second dose) (Table 2).

**Table 2:** Gestational age of enrolled subjects

Gestational age	Patients enrolled (747)	100%
First trimester	113	15.3%
Second trimester	123	16.5%
Third trimester	510	68.3%

Among 457 primigravida, 431 received the first dose, which is attributed to 94.4%, and 381 received the second dose, which is attributed to 83.3%. In multigravida, the first dose was received by 275 patients (94.7%), and 234 (80.7%) subjects received the second dose, which showed that there was no significant relationship between parity and vaccination (p=0.4541). None of the subjects took a booster dose of the COVID-19 vaccine (Table 3).

**Table 3:** Parity and vaccination

Gravida	n	1st dose	2nd dose	Booster dose	P value
Primigravida	457 (61.2%)	431 (94.4%)	381 (83.3%)	-	0.4541
Multigravida	290 (38.8%)	276 (94.7%)	224 (80.7%)	-	
Total	747	707 (94.6%)	705 (94.3%)	-	

Among the total of 747 subjects, 707 (94.6%) subjects received the first dose of the COVID-19 vaccine, whereas 40 (5.4%) subjects did not receive any dose. A total of 605 subjects received the second dose of the COVID-19 vaccine, among which 600 (80.3%) subjects received the vaccine before pregnancy, whereas only five (0.7%) women received the vaccine during pregnancy. A total of 142 (19%) subjects did not take the second dose of COVID-19 vaccine at all. None of the subjects took a booster dose of COVID-19 vaccine (Table 4).

**Table 4:** COVID-19 vaccine status of the enrolled subjects

Variable	Subdomain	n (%)
First COVID-19 vaccine dose	Taken before pregnancy	707 (94.6%)
	Pending	40 (5.4%)
Second COVID-19 vaccine dose	Taken before pregnancy	600 (80.3%)
	Taken during pregnancy	5 (0.7%)
	Pending	142 (19%)
Booster COVID-19 vaccine	Pending	747 (100%)

When PW were asked about vaccine hesitancy, different reasons and myths were stated by them. The commonest reason for not taking the COVID-19 vaccination was the fear of abortion in 179 (23.9%) subjects, followed by the fear of vaccine-related side effects in 142 (19%) subjects. The lack of resources and guidelines, awareness, and knowledge during pregnancy was cited by 86 (11.6%) and 76 (10.2%), respectively. About 10% of the women thought it would increase the risk of COVID-19 infection in PW. Thirty-five, or approximately 5% of women, had comorbidity. Twenty-five (3.4%) PW thought that COVID-19 can get transmitted to the fetus by vaccination, and the other 15 (2.1%) said that it may retard their baby’s growth. Five (0.7%) women did not take any dose of the vaccine. Twenty (2.8%) had the fear that vaccination could cause preterm labor. Five (0.7%) refused because of self-myths. Some of them were misguided by healthcare workers at the peripheral level, quacks, and family: five (zero point seven), 10 (1.4%), and 20 (2.8%), respectively (Table 5).

**Table 5:** Reason for noncompliance with the COVID-19 vaccine

S. No.	QUESTIONS	n (%)
Q1	Can cause abortion	179 (23.9%)
Q2	Fear of side effects	142 (19%)
Q3	Lack of resources/vaccine	86 (11.6%)
Q4	Lack of awareness about guidelines for COVID-19 vaccination	76 (10.2%)
Q5	Can increase the risk of COVID-19 infection in PW	74 (10%)
Q6	Mother had comorbidities	35 (4.7%)
Q7	Can cause COVID-19 in the fetus	25 (3.4%)
Q8	Can cause intrauterine fetal death	24 (3.2%)
Q9	Vaccination can cause preterm labor	20 (2.8%)
Q10	Source of information	
	Media and the internet	24 (15.56%)
	Family/friends	20 (2.8%)
	Quacks	10 (1.4%)
	Healthcare workers	5 (0.7%)
Q11	Can cause intrauterine growth retardation	15 (2.1%)
Q12	Side effects with the first dose	11 (1.5%)
Q13	Can cause a birth anomaly in the fetus	10 (1.4%)
Q14	Lactating	5 (0.7%)
Q15	Self-myth	5 (0.7%)

## Discussion

There are myths and doubts about any type of medicine/vaccine during pregnancy, including COVID-19 vaccination. False myths and beliefs have been heightened because of the spread of false information on social media, hearsay information, and incorrect clinical information about COVID-19 vaccination. There are many myths present across the globe concerning vaccines, especially during pregnancy. In the current study, we found that the mean age of the study subjects was 27.39 ± 3.75 years. We concluded that education and employment had a highly significant P value, which means that misconceptions, misinformation, and false beliefs related to COVID-19 vaccination can be prevented by raising the education level and providing employment. The study by Miral et al. had similar findings [8]. Thus, it can be said that education and employment can play a significant role in the awareness and acceptance of COVID-19 vaccination in general and in the pregnant population. It increases vaccination acceptance rates and makes it easier to reach the developments in the field of health and the right information sources. In our study, 162 women had not received vaccination, and most were homemakers. Similar results were found in the study done by Skjefte et al., where they also mentioned that employment has a strong correlation with acceptance of vaccination [9]. It explains that employed women are more in contact with the external environment, which increases their probability of infection, thereby increasing vaccine acceptance in working women. In our study, most patients were in the third trimester, followed by second and first trimester, which is in accordance with a study done by Egloff et al. The mean gestational age was 30 ± 5 weeks, and half of them were in the third trimester [10]. It might be because of the COVID-19 protective behavior guidelines issued by governments to avoid hospitals and crowded places until absolutely necessary. Hence, they were avoiding hospitals unless there was any type of emergency. In our study, the majority of the patients enrolled were primigravida, and a statistically nonsignificant relation was present between dose and parity. In contrast to our study, Naqvi et al. found that subjects hesitant to get vaccinated were multigravida (66.3%) [11]. The current study is a single-center study, while the study by Naqvi et al. was multicentric. The current study found that the proportion of subjects not receiving the first and second doses of vaccination was very small as they had received doses preconceptionally. A mere 0.6 percent received vaccination in pregnancy (second dose), but none of them received a booster dose. Noncompliance toward vaccination during pregnancy was most commonly because of the fear of abortion (23.9%), followed by the fear of side effects of the vaccine in 19% of the study population. Similarly, Berkowitz et al. found the misconception of abortion as a leading reason for noncompliance [12]. Various studies conducted in 2021 showed a similar risk of miscarriage irrespective of COVID-19 vaccination status [13,14]. Another 11.6% said that they lacked the resources/facility for vaccination, and (10.2%) of the population lacked awareness. Another 10% had the fear of vaccine-related COVID-19 infection. Hence, to improve the health status of PW by the vaccination drive for COVID-19, considerable educational effort is needed from health professionals to provide accurate information and guidelines regarding COVID-19 vaccination. Approximately 4.7% of people thought that the existing comorbidities may worsen with the vaccine. The previous studies at our institute in the general population concluded that comorbidities such as hypertension, diabetes mellitus, depression, immunity, and so on, predispose the person to a high risk for morbidity and mortality [15,16]. Vaccination is essential for them, and great care is needed so as not to get predisposed to COVID-19 infection. A study done by Lenin et al. concluded that maternal mortality was similar in the first and second waves, but perinatal mortality was more in the prevaccination era during the first and second waves [17]. Few subjects stated that it may harm their fetus in utero in the form of demise (3.2%), preterm labor (2.8%), growth retardation (2.1%), and congenital anomaly (1.4%). A study conducted by Janik et al. concluded that insufficient knowledge of the effects or complications of the vaccine in the fetus was the reason for refusal [18]. Women received misguidance from various sources (20.46%), such as media and the internet (15.56%), family/friends (2.8%), quacks (1.4%), and healthcare workers (0.7%). Uludağ et al. [19] from Turkey found that most of the PW experienced COVID-19 vaccine hesitancy as they were doubtful of the vaccine’s safety for their fetuses, and the reason behind this was social media, mass media, misguidance by health professionals, negative behavior about the vaccine from their family members, friends. Only a few (0.7%) women conceived of lactational amenorrhea, so they refused the vaccine, and another 0.7% had their own myths, and they did not cite any reason for noncompliance with COVID-19 vaccination. Approximately 1.5% of PW did experience side effects, such as high-grade fever, myalgias, and pain at the injection site with the first dose of the vaccine, resulting in the denial of the second dose/booster. Shimabukuro et al. found that injection-site pain was more frequent in PW, while headache, myalgia, chills, and fever were reported less frequently [20]. These side effects are mild and can be decreased with paracetamol. The major limitation of our study was that it was a single-center study; therefore, the outcome cannot be generalized for the entire local population, and a larger sample size would have provided more significant findings.

## Conclusions

The present study was a prospective study conducted to analyze the myths present in PW related to the COVID-19 vaccination. There is a significant correlation between vaccination, employment, and education. Education and employment may help increase PW’s confidence in COVID-19 vaccine safety and accelerate vaccine uptake. We believe that education campaigns focusing on PW are needed. Healthcare workers play an important role in improving the coverage of the COVID-19 vaccine by providing resources and sufficient knowledge about the effectiveness and safety of the vaccine even during pregnancy and lactation. Social media platforms should improve information regulation by modifying community standards, implementing surveillance algorithms, and applying warning labels to potentially misleading posts.

## References

[1] Badell ML, Dude CM, Rasmussen SA, Jamieson DJ (2022). Covid-19 vaccination in pregnancy. BMJ.

[2] Burki T (2020). The indirect impact of COVID-19 on women. Lancet Infect Dis.

[3] Lamptey E (2022). Overcoming barriers to COVID-19 vaccination of pregnant women. Gynecol Obstet Clin Med.

[4] Kumari U, Sharma RK, Sinha A, Sinha M, Keshari JR (2023). Impact of COVID-19 vaccination on women during pregnancy and breastfeeding. Cureus.

[5] Gupta A, Christina S, Umar AY, Laishram J, Akoijam BS (2022). COVID-19 vaccine hesitancy among pregnant women: a facility-based cross-sectional study in Imphal, Manipur. Indian J Public Health.

[6] Bednarek A, Laskowska M (2024). Vaccination guidelines for pregnant women: addressing COVID-19 and the omicron variant. Med Sci Monit.

[7] Martins I, Louwen F, Ayres-de-Campos D, Mahmood T (2021). EBCOG position statement on COVID-19 vaccination for pregnant and breastfeeding women. Eur J Obstet Gynecol Reprod Biol.

[8] Miral MT, Turgut N, Güldür A, Güloğlu ZE, Mamuk R (2023). COVID-19 fear, vaccination hesitancy, and vaccination status in pregnant and breastfeeding women in Turkey. Afr J Reprod Health.

[9] Skjefte M, Ngirbabul M, Akeju O, Escudero D, Hernandez-Diaz S, Wyszynski DF, Wu JW (2021). COVID-19 vaccine acceptance among pregnant women and mothers of young children: results of a survey in 16 countries. Eur J Epidemiol.

[10] Egloff C, Couffignal C, Cordier AG (2022). Pregnant women's perceptions of the COVID-19 vaccine: a French survey. PLoS One.

[11] Naqvi S, Saleem S, Naqvi F (2022). Knowledge, attitudes, and practices of pregnant women regarding COVID-19 vaccination in pregnancy in 7 low- and middle-income countries: an observational trial from the Global Network for Women and Children's Health Research. BJOG.

[12] Berkowitz HE, Vann JC (2023). Strategies to address COVID-19 vaccine and pregnancy myths. MCN Am J Matern Child Nurs.

[13] Magnus MC, Gjessing HK, Eide HN, Wilcox AJ, Fell DB, Håberg SE (2021). Covid-19 vaccination during pregnancy and first-trimester miscarriage. N Engl J Med.

[14] Zauche LH, Wallace B, Smoots AN (2021). Receipt of mRNA Covid-19 vaccines and risk of spontaneous abortion. N Engl J Med.

[15] Naseem N, Thakur R, Sadhu R (2022). Presentation, clinical course and outcome in hospitalized patients with Covid - 19: a retrospective study. Alq J Med App Sci.

[16] Kaur G, Vidyalakshmi S, Sadhu R (202212). A retrospective study on COVID-19 in a tertiary care rural hospital. Natl J Physiol Pharm Pharmacol.

[17] Lenin A, Abraham K, David LS, Tirkey RS, Mani T, Jasmine S, Sathyendra S (2023). Comparison between the demographic shift clinical severity and outcome of the first two waves of COVID-19 in pregnancy in a tertiary hospital in India. Int J Gynaecol Obstet.

[18] Janik K, Nietupska K, Iwanowicz-Palus G, Cybulski M (2022). Fear of COVID-19 and vaccine hesitancy among pregnant women in poland: a cross-sectional study. Vaccines (Basel).

[19] Uludağ E, Serçekuş P, Yıldırım DF, Özkan S (2022). A qualitative study of pregnant women's opinions on COVID-19 vaccines in Turkey. Midwifery.

[20] Shimabukuro TT, Kim SY, Myers TR (2021). Preliminary findings of mRNA Covid-19 vaccine safety in pregnant persons. N Engl J Med.

